# A SPACE OF OUR OWN: RECLAIMING PUBLIC OPEN SPACES FOR SELF-EXPRESSION AND COMMUNITY

**DOI:** 10.1093/geroni/igae098.0020

**Published:** 2024-12-31

**Authors:** Ad Maulod, Malcolm Ravindran, Yunjie Wong

**Affiliations:** Duke-NUS Medical School, Singapore, Singapore; Duke-NUS Medical School, Singapore, Singapore; Duke-NUS Medical School, Singapore, Singapore

## Abstract

Public open spaces give older persons opportunities to build social capital and community, and are important sites in promoting self-expression, meaning- and place-making– attributes crucial to successful ageing-in-place. In Singapore, residents have little influence over the design and production of public open spaces, while regulations on the use of these spaces may dissuade utilization by older persons. Despite ‘age-friendly’ principles undergirding Singapore’s built environment, contestations arise in terms of top-down planned activity-usage, and older persons’ actualization of what aspirations they have in using such spaces. Recently, the government has increased the provision of ‘active ageing centres’ nationwide, intended to be inclusive, low barrier-to-entry spaces within residential neighbourhoods, to encourage social engagement and community support among older persons. However, older persons’ perceptions of designated senior-spaces as catering to the sick and vulnerable, leads to hesitance in utilizing those spaces. Drawing from ethnographic interviews across multiple qualitative research studies with over 200 older persons in Singapore, we found that participants expressed a lack of control over their community spaces, programme offerings, and are not empowered to organize activities with fellow residents. Yet, their narratives also highlight creative strategies to resist regulatory practices and inhabit public open spaces with activities that align with their desires to age well. Public open spaces are crucial to older persons’ freedom and autonomy. Accessibility, in terms of physical and social infrastructure, needs to be reframed in ways that value older persons’ diverse interests, capacity to self-organize, and potential for building community.

